# Analysis of the Fiber Residues Unearthed from the Dabuzi Han Tomb in Xi’an, Shaanxi

**DOI:** 10.3390/ma18204812

**Published:** 2025-10-21

**Authors:** Zhenzhen Ma, Yingpei Zhu, Jing Shao, Xianting Hou, Menghe Cui, Bei Zhang, Jianxi Li, Qixing Xia

**Affiliations:** 1 Shaanxi Academy of Archaeology, Xi’an 710109, China; mazhenhappy1990@163.com (Z.M.);; 2 Key Scientific Research Base of On-Site Conservation, State Administration for Cultural Heritage, Xi’an 710109, China; 3 Shaanxi Key Laboratory of Archaeological Conservation, Xi’an 710109, China; 4 Shaanxi History Museum, Xi’an 710061, China; 5 Institute of Culture and Heritage, Northwestern Polytechnical University, Xi’an 710072, China; 6 Key Laboratory of Archaeological Exploration and Cultural Heritage Conservation Technology (Northwestern Polytechnical University), Ministry of Education, Xi’an 710129, China

**Keywords:** fiber residues, microscopic analysis, FTIR, Py-GC/MS, fixed-mold method

## Abstract

In 2021, archeologists found that a bronze mirror was wrapped with a yellow-green fiber sheet in the Western Han tomb M68 in the Dabuzi Cemetery in Xi’an, China. To ascertain the composition and function, a scanning electronic microscopy–energy dispersive spectrometer (SEM-EDS), Fourier transform infrared spectroscopy (FTIR), and pyrolysis gas chromatography–mass spectrometry (Py-GC/MS) were combined for the morphology and components’ analysis. The results showed that the surface of the yellow-green fiber sheet was very rough without curtain patterns, and the fiber was disorderly intertwined. The paper was quite thick with various thicknesses (the average thickness was 0.58 mm) and the average diameter of the fiber was 20.71 μm. There were obvious transverse joint stripes on the fiber cell with longitudinal stripes characteristic of ramie or hemp. The main ingredients were cellulose, semi-cellulose, and lignin. Based on the above comprehensive joint experiments, the yellow-green fiber sheet in M68 was presumably ancient hemp paper made with the fixed-mold method. Moreover, it was speculated to be a package material since no characters were found. This paper is of great significance for studying the Chinese fixed-mold paper-making technique and for understanding the origins and developmental trajectory of ancient paper-making technology.

## 1. Introduction

**Figure 1:**
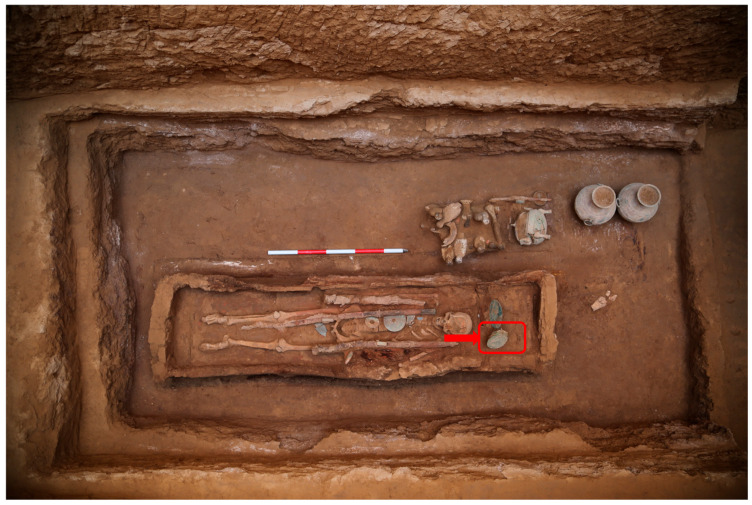

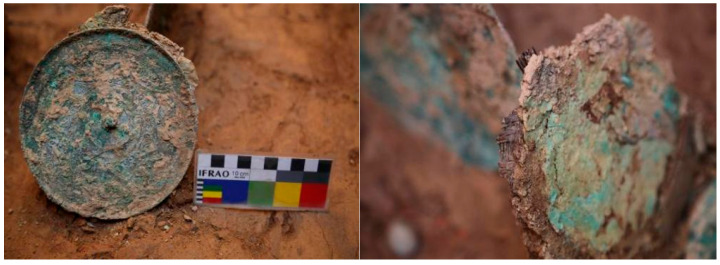
The excavation site of the yellow-greenish fiber sheet on the bronze mirror (the excavation location of the mirror was indicated by the red box and arrow).

The Dabuzi Cemetery is located on the terrace south of the Jing River (northeast of the Xianyang Plateau) in Xi’an China. Since 2020, the Shaanxi Academy of Archaeology have excavated and cleaned over 400 tombs, unearthing nearly 2000 artifacts (groups) made of pottery, bronze, jade, iron, and other materials, primarily dating to the early Western Han Dynasty. Among these, Tomb M68, a vertical shaft tomb with an earthen chamber, stood out as the most well-preserved, yielding the largest quantity, richest variety, and most complete assemblage of artifacts discovered at the Dabuzi site. The tomb contained precious cultural relics, including gilded bronze objects, bronze vessels, jade artifacts, large painted pottery granaries, and sets of painted pottery figurines depicting music and dance performances. A jade seal from the burial chamber identified the tomb occupant as “Yao She.” Two bronze mirrors were found near the head of the deceased, one of which was attached to yellow-green fibrous sheets suspected to be paper (Figure 1). However, determining whether this material was paper from the Western Han Dynasty required systematic scientific analysis.

Currently, non-destructive and minimally invasive analytical techniques for paper artifacts are highly advanced, including a scanning electronic microscopy–energy dispersive spectrometer (SEM-EDS), atomic force microscopy (AFM), confocal Raman microscopy, X-ray photoelectron spectroscopy (XPS), fiber-optic reflectance spectroscopy (FORS), terahertz spectroscopy, and micro-computed tomography (micro-CT). Functionally, these techniques could be categorized into imaging methods—such as optical, infrared, ultraviolet, X-ray, optical microscopy, SEM-EDS, and transmission electron microscopy (TEM)—which reveal microscopic structural information, and spectroscopic methods—including chromatography, mass spectrometry, nuclear magnetic resonance (NMR), X-ray fluorescence (XRF), molecular fluorescence spectroscopy, and Raman spectroscopy—which provide compositional insights [1,2,3,4,5,6]. Meanwhile, minimally invasive pyrolysis gas chromatography mass–spectrometry (Py-GC/MS) and Fourier transform infrared spectroscopy (FTIR) were widely used in the organic residues’ composition identification [7,8,9].

In this study, we employed stereo microscopy, SEM-EDS, FTIR, and Py-GC/MS to analyze the yellow-green fibrous sheet wrapped around the bronze mirror from Tomb M68. A combined approach of morphological and compositional analysis was adopted; preliminary characterization of the sample’s physical properties was conducted through microscopic examination, followed by the precise determination of its chemical composition using FTIR and Py-GC/MS. This research holds significant importance for advancing our understanding of ancient Chinese paper-making technology.

## 2. Materials and Methods

### 2.1. Sampling

During the archeological excavation, surface soil covering the bronze mirror and paper-like residue were carefully removed. A black-brown fibrous woven material was discovered adhered to the reverse side of the paper-like residue. Using tweezers, two samples were gently separated and collected, each of them was stored in individual specimen containers.

### 2.2. Instrumental Analysis

#### 2.2.1. Morphological Observation

(1) Ultra-Depth Microscopy Observation
  Portions of the yellow-green fibrous sheet and individual black-brown fibers were extracted and examined under a Zeiss Smartzoom 5 ultra-depth microscope (Germany, Oberkochen, Zeiss). Microscopic images were captured at magnifications of 50×, 100×, 200×, and 300×.
(2) Biological Microscopy Observation
  Following the GB/T 4688-2002 standard [10], small quantities of the two aforementioned samples were placed in stoppered test tubes. A hydrogen peroxide–glacial acetic acid solution (1:1 by volume) was added, and the mixture was incubated in a 60 °C water bath for 24 h. The samples were then filtered through a 300-mesh chemical fiber bag and rinsed. A small amount of the treated sample was placed on a glass slide, stained with 1–2 drops of dye, and evenly dispersed using a dissecting needle. A coverslip was applied, and excess dye was gently absorbed by tilting the slide and using absorbent paper. Observations and imaging were performed under a Leica DM4000B biological microscope (Germany, Wetzlar, Leica Microsystems).
(3) SEM-EDS Observation and Analysis
  The structural features of the yellow-green fibers were examined using a JEOL JSM-7500F field-emission scanning electron microscope (Japan, Tokyo, JEOL Ltd.) with a X-Max 50 EDS (England, Oxford, Oxford Instruments). SEM testing conditions included sample coating: ~10 nm of 99.99% gold; accelerating voltage: 5 kV; beam current: 1 × 10^−10^ A; and working distance: 8 mm. EDS testing conditions were as follows: accelerating voltage: 15 kV; beam current: 3 × 10^−10^ A; and working distance: 8 mm.

#### 2.2.2. FTIR Analysis

A Nicolet iN10 FTIR microscope (equipped with a Nicolet iZ10™ FTIR auxiliary optical stage, Madison, WI, USA, Thermo Fisher Scientific) was used for infrared testing. A small amount of the powdered sample was placed on a BaF_2_ window for transmission mode analysis, with the BaF_2_ window serving as the background. The MCT/A detector (Madison, WI, USA, Thermo Fisher Scientific) was employed under the following parameters: spectral range: 650–4000 cm^−1^; resolution: 4 cm^−1^; and scans: 32.

#### 2.2.3. Py-GC/MS Analysis

Instrumentation and conditions: The pyrolyzer was a Frontier Lab PY-3030D (Japan, Fukushima-shi, Frontier Laboratories Ltd.) the gas chromatograph/mass spectrometer was a Shimadzu GC/MS-QP2010 Ultra (Japan, Kyoto, Shimadzu Corporation); column: DB-5MS (frontier lab; stationary phase: 5% diphenyl dimethyl polysiloxane, Santa Clara, CA, USA, Agilent Technologies), length: 30 m, inner diameter: 0.25 mm, and film thickness: 0.25 μm. Pyrolysis conditions: temperature: 550 °C, duration: 12 s, injector temperature: 280 °C, and GC/MS interface temperature: 300 °C. Gas chromatography program: initial temperature: 50 °C (hold for 5 min), ramp rate: 5 °C/min to 300 °C (hold for 10 min). MS parameters: carrier gas was high-purity helium, inlet pressure: 100 kPa, split ratio: 1:20. The electronic pressure control system operated in constant current mode, Electron ionization (EI) energy: 70 eV; mass range (*m*/*z*): 30–750; cycle time: 0.5 s; and spectral libraries: NIST17 and NIST17s [11].

Method: Approximately 0.1 mg of sample was weighed into a pyrolysis-specific sample cup, which was then loaded into the pyrolyzer’s auto-sampler. The Py-GC/MS system was initiated according to the predefined method.

## 3. Results and Discussion

### 3.1. Morphological Observation Analysis

**Figure 2:**
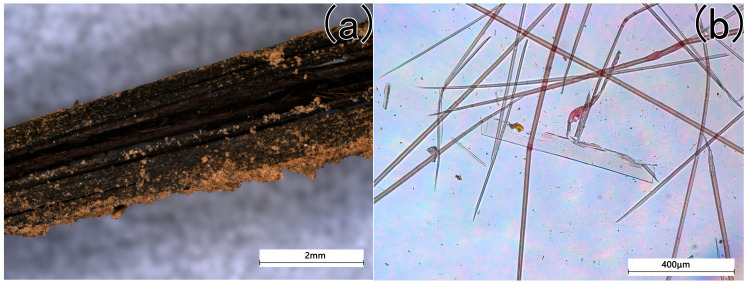
The microscopical pictures of the strip fiber (**a**) before chemical treatment, (**b**) after chemical treatment).

**Figure 3:**
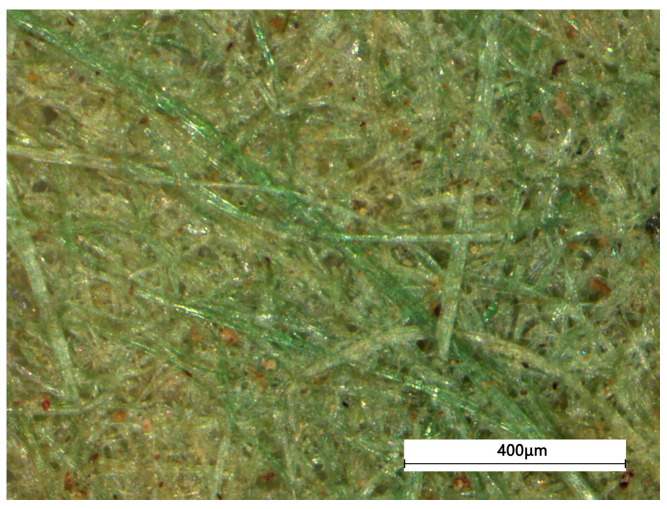
The microscopical pictures of the yellow-greenish fiber.

The microscopic morphology of the black-brown fibers and yellow-green fibrous sheet are shown in Figure 2 and Figure 3. As shown in Figure 2a, the individual black-brown fibers exhibit a smooth surface and intact edges, indicating an excellent conservation state. After chemical treatment, the samples show the appearance of stiff slender filaments. Under biological microscopy, the fibers display an elongated morphology with rectangular vessel cells (Figure 2b), consistent with the characteristic features of bamboo fibers [12]. Combined with their archeological context and the historical habit of bamboo mats for wrapping objects, the black-brown residue is identified as a woven bamboo artifact.

Figure 3 presents the initial ultra-depth microscopy observations, the blue-green coloration is typical of bronze corrosion products, while the yellow represents the natural color of the fibers. According to the SEM-EDS results of the fiber (Table 1), the variegated appearance is attributed to the prolonged contact between the hemp fibers and the bronze mirror (41.47% Cu). The yellow-green fibrous sheet displays a rough intact surface with multiple fibers randomly interwoven. No laid lines—a hallmark of Eastern Han paper-making techniques attributed to Cai Lun—are observed, ruling out the movable-mold paper-making method.

**Figure 4:**
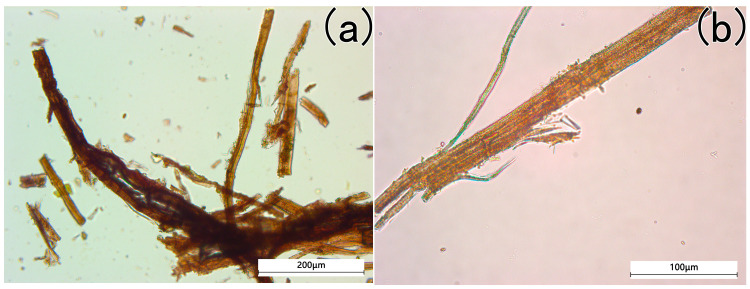
The microscopical pictures of the yellow-greenish fiber after chemical treatment (**a**) slight fibrillation at the fiber edges, (**b**) ramie or hemp fibers.

After treatment, detailed biological microscopy analysis (Figure 4a) reveals slight fibrillation at the fiber edges, indicating that manual beating occurred during processing. Additionally, transverse nodes and longitudinal striations, distinctive features of ramie or hemp fibers are observed on the cell walls of some fibers in the yellow-green sheet (Figure 4b). These findings confirm the presence of bast fibers in the sample, aligning with the compositional profile of Han Dynasty paper [13].

**Figure 5:**
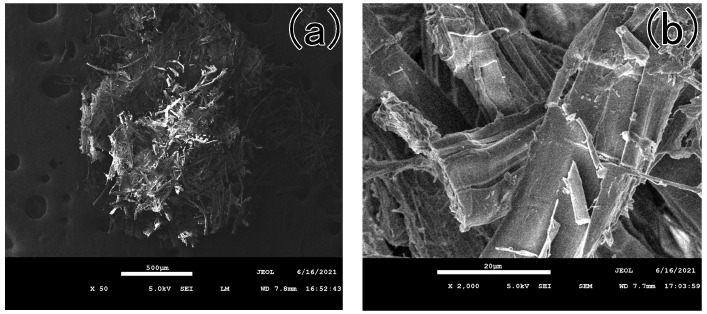
The SEM pictures of the yellow-greenish fiber (**a**) the disordered and loosely aggregated state of the fibrous sheet, (**b**) the cutting marks on the fibers.

SEM images reveal that the yellow-green fibrous sheet consisted of numerous short fibers exhibiting a disordered and loosely aggregated state (Figure 5a). This morphological arrangement markedly differs from the unidirectional natural structure characteristic of raw hemp fibers. Furthermore, distinct cutting marks are observed on the fibers (Figure 5b), providing additional evidence that the fibrous sheet underwent intentional cutting during artificial processing.

**Figure 6:**
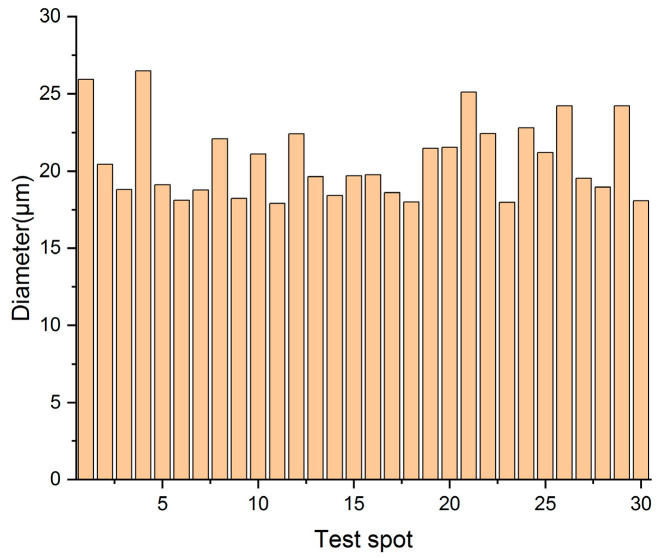
The diameters of the yellow-greenish fiber.

**Figure 7:**
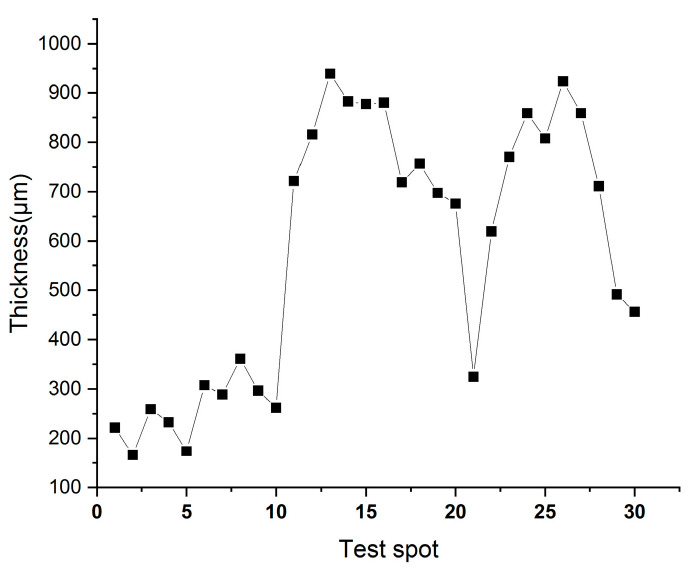
The thickness of the yellow-greenish paper.

Three distinct areas of the yellow-green fibrous sheet are examined and photographed using ultra-depth microscopy, 10 fiber diameters are measured at each location (Figure 6). Cross-sectional morphology is similarly documented at three representative locations, with thickness measured at 10 points per spot (Figure 7). The fibers show an average diameter of 20.71 μm (range: 17.91–26.49 μm), closely matching published data for hemp paper fibers [14]. Comparative measurements of hemp (20.87 μm), ramie (27.86 μm), and flax (17.06 μm) [15] suggest the material was most likely hemp, given its predominant diameter around 20 μm. Notably, the sheet exhibits significant thickness variation (0.17–0.94 mm, average: 0.58 mm), indicating relatively primitive manufacturing techniques.

Based on the aforementioned morphological analysis, it could be concluded that the black-brown residue is bamboo-woven wrapping material, while the yellow-green fibrous flakes exhibit signs of artificial processing and might represent ancient paper that predates the use of the movable-mold paper-making method. Beyond these morphological characteristics, further compositional analysis is required to verify further findings.

### 3.2. FTIR Discussion

**Figure 8:**
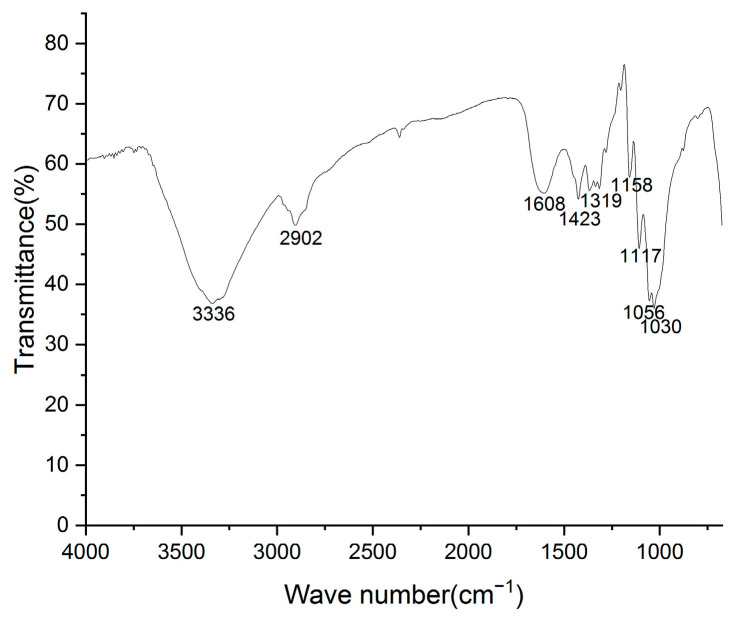
The FTIR spectrum of the yellow-greenish fiber.

The infrared spectrum of the yellow-green fibrous sheet (Figure 8) exhibits characteristic absorption bands that are consistent with typical paper spectra [16]. The observed peaks include the following: 3336 cm^−1^ (O-H stretching vibration of cellulose), 2902 cm^−1^ (C-H stretching vibration of cellulose), 1608 cm^−1^ (C=O stretching vibration of aromatic rings in lignin), 1423 cm^−1^ (symmetric bending vibration of -CH_2_ at the C-6 position of cellulose), 1319 cm^−1^ (-CH_2_ wagging vibration at the C-6 position of cellulose), 1158 cm^−1^ (C-O-C glycosidic bridge stretching vibration of β-linkages in cellulose/hemicellulose), 1117 cm^−1^ (ring stretching vibration of cellulose), 1056 cm^−1^ (C-OH stretching vibration of cellulose), and 1030 cm^−1^ (C-O stretching vibration at the C-6 position of cellulose) [17]. These characteristic absorption peaks clearly demonstrated that the sheet was primarily composed of cellulose, hemicellulose, and lignin, which matched the chemical composition of paper materials.

### 3.3. Pyrolysis Gas Chromatography–Mass Spectrometry (Py-GC/MS) Analysis

**Figure 9:**
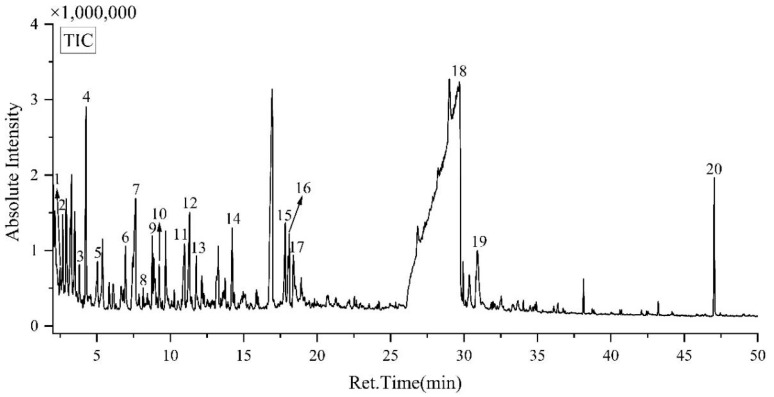
The Py-GC/MS TIC chromatogram of the yellow-greenish fiber.

The Py-GC/MS analysis of the yellow-green fibrous sheet (Figure 9 and Table 2) revealed that pyrolysis products could be categorized into four main groups. To be specific, (1) Furan substances: 1 and 2; (2) aldehydes and ketones: 3, 4, 5, 6, 7, 8, 9, 11, 12, 13 and 17; (3) phenol substances: 14 and 20; and (4) sugar substances: 15, 16, 18, and 19. Among these compounds, the sugar substances (4), which are characteristic pyrolysis products of cellulose, show the highest relative abundance, indicating cellulose as the predominant component in the sample. The aldehydes and ketones (2) are typical degradation products of both hemicellulose and cellulose, while the phenolic compounds (3) mainly originate from lignin. These three components (cellulose, hemicellulose, and lignin) collectively represent the fundamental chemical constituents of paper materials which are in accordance with the Pyrolysis Characterization of Nepalese Handmade Paper [18]. The Py-GC/MS results thus provide molecular-level confirmation of the sheet’s paper-like composition, corroborating the findings from the other analytical techniques [7,8].

### 3.4. Discussion of the Sample

Archeological evidence demonstrated that plant fiber-based hemp paper was already invented during the early Western Han Dynasty in China, as exemplified by several significant discoveries [19]. The Baqiao paper, unearthed in the Baqiao District, Xi’an in 1957, was made solely from hemp bast fibers and appeared as fragmented light-yellow sheets upon excavation, exhibiting uneven fiber distribution while maintaining reasonable structural integrity [20]. Another notable finding was the Zhongyan paper discovered in 1978 in Fufeng County, Shaanxi Province, which displayed a rough surface with wrinkles and variable fiber thickness (averaging 0.277 mm), showing clear signs of mechanical beating through characteristic fiber fibrillation [21]. In the neighboring Gansu Province, important discoveries included the Xuanquanzhi paper, which has predominantly yellow uneven, rough sheets with chaotic fiber arrangements illustrating low-beating-degree pulp [22], and the Jinguan paper which consisted of thick, uneven sheets with even bast fibers resembling the Zhongyan paper in texture [23].

These early papers consistently shared three distinctive features characteristic of the fixed-mold paper-making technique: significant thickness, absence of laid lines, and rough surfaces with disordered fiber orientation [23]. Professor Li Xiaocen’s research revealed that the fixed-mold paper-making method utilized raw materials such as hemp, mulberry bark, and paper mulberry bark. These materials underwent cleaning and were then boiled in iron pots with stove ash for alkalization treatment, followed by pulping using wooden mallets or stones. The processed pulp was poured onto a fixed screen placed horizontally on water’s surface, with both the screen and paper being sun-dried together before paper separation [24]. This method typically produced thick paper without the characteristic screen marks of bamboo-screen paper making and exhibited a rough surface texture due to the absence of pressing. Microscopic examination revealed randomly oriented fibers resulting from the top-down pouring process. In the 1970s, Taiwanese paper history scholar Chen Dachuan identified two distinct paper-making techniques: the fixed-mold paper-making method and the movable-mold paper-making method, through investigations of traditional paper-making in Southeast Asia, Professor Li’s extensive field research and laboratory experiments subsequently established these as two separate paper-making systems preserved in mainland China [25], with historical origins tracing to the Western Han Dynasty for the fixed-mold paper-making method and the Eastern Han Dynasty for the movable-mold paper-making method. Contemporary practice showed that while most Han Chinese and ethnic minority groups employed the movable-mold paper-making method, several minority communities including the Dai and Naxi in Yunnan, Uyghurs in Xinjiang, Tibetans in Sichuan and Tibet, and the Dong in Guizhou provinces continued to preserve the fixed-mold paper-making method [26]. Fixed-mold paper making’s production efficiency remained limited by its “one-screen-one-sheet” constraint, requiring complete drying before sheet removal. In contrast, the movable-mold paper-making’s multi-step process (including peeling, retting, boiling, pounding, and bleaching) and “one-screen-multiple-sheets” approach significantly enhanced productivity, leading to its widespread adoption, as recorded in historical texts. This technically advanced method had maintained continuous production for nearly two millennia up to the present day.

Furthermore, early Western Han Dynasty paper may not have been primarily used for writing purposes. As recorded in the Hanshu Biography of Empress Zhao of Xiaocheng: “In the box were two packets wrapped in ‘Heti’, containing a written secret order.” This described how Consort Zhao used “Heti” (thin, small paper) to wrap two doses of poison while also inscribing a secret command in her attempt to poison Cao Weineng, a palace maid favored by Emperor Cheng of Han [27]. The commentator Ying Shao noted that “Heti referred to thin, small pieces of paper.”

Based on the experimental results from Section 3.1 to Section 3.3, the yellowish-green fibrous sheet (M68) discussed in this study exhibited several distinctive characteristics: it had uneven thickness (averages 0.58 mm) which was twice that of Zhongyan paper (0.277 mm) produced by the fixed-mold making method; the surface was rough with chaotic, disordered fiber distribution and showed cutting marks; furthermore, no screen marks were observed. These features are entirely consistent with the characteristics of paper made by the fixed-mold making method. Additionally, some fibers displayed distinct transverse nodes and the characteristic longitudinal striations of hemp fibers, with the average diameter suggesting Cannabis sativa (hemp) as the probable material. FTIR and Py-GC/MS analysis confirmed the main components as cellulose, hemicellulose, and lignin. As no writing appeared on its surface, it served solely as wrapping material for a bronze mirror. In conclusion, we preliminarily identified the yellowish-green fibrous sheet unearthed from the Han Dynasty tomb at Dabuzi, Xi’an, Shaanxi Province as hemp paper manufactured during the Western Han Dynasty using the fixed-mold making method, specifically for wrapping purposes.

## 4. Conclusions

(1) Combined observations of ultra-depth microscopy, biological microscopy, and scanning electron microscopy revealed that the yellowish-green sheet unearthed from the Han Dynasty tomb at Dabuzi, Xi’an, Shaanxi Province exhibited a rough surface with randomly distributed fibers, showing evidence of shearing and slight fibrillation, which are clear indications of manual beating and cutting processes during production. Its considerable and uneven thickness, along with the absence of screen marks on the surface, are characteristic features consistent with the fixed-mold paper-making technique.
(2) Microscopic examination identified distinct transverse nodes on some fiber cell walls, along with longitudinal striations characteristic of either ramie or hemp fibers. With an average fiber diameter of 20.71 μm, the material was most probably hemp (Cannabis sativa). Complementary analyses using FTIR and Py-GC/MS confirmed cellulose, hemicellulose, and lignin, as the principal components of the paper.
(3) Comprehensive analysis led to the preliminary conclusion that this yellowish-green fibrous sheet represented hemp paper residue manufactured by the fixed-mold making method. Dating based on the tomb’s chronology placed it in the Western Han Dynasty (206 BCE-9 CE). The absence of surface inscriptions suggested its primary function was wrapping material for bronze mirrors. This discovery and its subsequent study hold significant implications for understanding the origins and developmental trajectory of ancient paper-making technology.

## Figures and Tables

**Table 1:** The element content (wt.%) of the yellow-greenish fiber via SEM-EDS.

Element	wt.%
C	18.47
O	33.35
Al	2.58
P	4.14
Cu	41.47
Total	100.00

**Table 2:** The Py-GC/MS result of the yellow-greenish fiber.

No.	Retention Time (min)	Peak Area (%)	Compounds
1	2.48	0.41	2,3-Dihydro-3-methylfuran
2	2.67	0.87	2-methylfuran
3	3.80	0.41	furfural
4	4.26	2.71	furfural
5	5.04	0.44	2-butanone
6	6.95	0.86	2(5H)-Furanone
7	7.64	2.68	2-methylcyclopentanone
8	8.15	0.21	3-Methyl-2,5-furandione
9	8.77	0.86	5-methyl-2-furanyl-ethanone
10	9.24	0.42	3-Methyl-2,5-furandione
11	10.97	1.44	2-Hydroxy-3-methyl-2-cyclopenten-1-ketone
12	11.31	1.7	3-methyl-1,2-cyclopentanedione
13	11.77	0.53	4-Methyl-5H-furan-2-ketone
14	14.22	1.08	maltol
15	17.82	1.01	1,4:3,6-Dihydro-α-d-glucopyranose
16	18.02	1.34	2,3-dehydro-d-galactose
17	18.38	0.84	5-Hydroxymethylfurfural
18	29.00	78.86	1,6-dehydro-β-D-glucopyranose
19	30.90	1.78	1,6-dehydro-β-D-glucopyranose
20	47.04	1.55	2-((2H-benzotriazole)-2-yl)-4-(1,1,3,3-tetramethylbutyl) phenol

## Data Availability

The original contributions presented in this study are included in the article. Further inquiries can be directed to the corresponding authors.
